# MicroRNA-125b-5p suppresses *Brucella abortus* intracellular survival via control of A20 expression

**DOI:** 10.1186/s12866-016-0788-2

**Published:** 2016-07-29

**Authors:** Ning Liu, Lin Wang, Changjiang Sun, Li Yang, Wanchun Sun, Qisheng Peng

**Affiliations:** 1 Central Laboratory, The Second Hospital of Jilin University, Changchun, 130041 China; 2 Key Laboratory for Zoonosis Research, Ministry of Education, Institute of Zoonosis, Jilin University, Changchun, 130062 China; 3 College of Veterinary Medicine, Jilin University, Changchun, 130062 China

**Keywords:** Brucella abortus, MiR-125, A20, Macrophage

## Abstract

**Background:**

*Brucella* may establish chronic infection by regulating the expression of miRNAs. However, the role of miRNAs in modulating the intracellular growth of *Brucella* remains unclear.

**Results:**

In this study, we show that *Brucella. abortus* infection leads to downregulation of miR-125b-5p in macrophages. We establish that miR-125b-5p targets A20, an inhibitor of the NF-kB activation. Additionally, expression of miR-125b-5p decreases A20 expression in *B. abortus*-infected macrophages and leads to NF-kB activation and increased production of TNFα. Furthermore, *B. abortus* survival is attenuated in the presence of miR-125b-5p.

**Conclusions:**

These results uncover a role for miR-125b-5p in the regulation of *B. abortus* intracellular survival via the control of A20 expression.

## Background

The genus *Brucella* is responsible for the worldwide Brucellosis, a disease of domestic and wild animals which is transmissible to humans [1]. The main symptoms include chills, fever, anorexia, and arthritis in humans and abortion in infected animals, which develops into a chronic infection if not treated [1].

Brucellae are intracellular pathogens that have the ability to survive and multiply inside professional phagocytic cells. The success of *Brucella* as an intracellular pathogen depends largely on its ability to avoid the activation of host macrophages upon infection [2, 3]. *Brucella* reprograms the host macrophage transcriptome by suppressing the expression of activation-related genes [4]. Noncoding RNAs, including microRNAs (miRNAs), may play an important role in this process through post-transcriptional regulation [5]. Diverse biological activities, including cell activation, are regulated by miRNAs [6]. In particular, miRNA expression changes have been described during macrophage infection with *Brucella*, suggesting that miRNAs are likely to influence the bacteria intracellular growth [7].

MiRNAs are single-stranded RNAs (approximately 22 nucleotides in length) that post-transcriptionally regulate gene and/or protein expression [8]. MiRNAs are species, cell, and context specific, and are dynamically regulated as a function of time in response to environmental triggers. A specific host miRNA response has been documented in the context of infection with intracellular bacteria, such as *mycobacteria*, *Listeria monocytogenes*, *Francisella tularensis*, and *Salmonella enterica* [9]. Nevertheless, to our knowledge, there has been no research to indicate that a specific host miRNA regulates *Brucella* intracellular survival.

The A20 protein, encoded by *TNFAIP3*, is an inhibitor of inflammation and is induced in many cell types by a wide range of stimuli [10]. It has been most intensively studied as an inhibitor of NF-kB activation and plays a key role in the inhibition of inflammation and innate immunity [11]. Recent studies have shown that miRNAs, such as miR-125a, miR-125b, and miR-873, can activate the NF-kB pathway by targeting the expression of A20 [6, 12]. Additionally, in *Mycobacterium tuberculosis*-infected macrophages, downregulation of miR- let-7f was shown to enhance the expression of its target A20 [13], thereby attenuating inflammatory signaling and facilitating bacterial survival. Our previous study indicated that A20 promotes *Brucella* intracellular growth via inhibition of macrophage cell death and activation [14]. Considering that *Brucella* infection induces significant changes in miRNAs expression in macrophages [7], we have investigated whether miRNAs also participate in the regulation of *Brucella* growth by targeting A20 expression.

In this study, we report that the miR-125b-5p-mediated regulation of A20 (TNFAIP3) plays an important role in tuning the activation of *B. abortus*-infected macrophages. We demonstrate that the downregulation of miR-125b-5p during *B. abortus* infection enhances the expression of the A20 protein, thereby inhibiting NF-kB activation, and facilitating bacterial intracellular survival.

## Methods

### Materials

Anti- iNOS antibody was purchased from R&D Systems (Minneapolis, MN, USA). Anti- ERK and calnexin were purchased from Cell Signaling Technology (Danvers, MA, USA). Anti-A20, β-actin, IkBα antibodies and Polybrene and puromycin were purchased from Santa Cruz Biotechnology (Dallas, Texas, USA). Enzyme-linked immunosorbent assay (ELISA) kit for TNFα measurement was purchased from eBioscience (San Diego, CA, USA).

### Mice

C57BL/6 mice were obtained from commercial vendors and maintained under specific pathogen-free conditions in the animal facility of the Central Laboratory, The Second Hospital of Jilin University. The animal protocol was reviewed and approved by the Jilin University Institute Animal Care and Use Committee. The present investigations conform to the Guide for the Care and Use of laboratory Animals published by the US National Institutes of Health (NIH Publication No. 85–23, revised 1996).

### *Brucella* and macrophage cell line

The *Brucella abortus*, strain 2308, used in this study, was originally obtained from Ding’s laboratory at the China Institute of Veterinary Drug Control (Beijing, China) and was grown either in tryptic soy broth or on tryptic soy agar plates. The RAW264.7 macrophage and HEK293T cell lines were purchased from the American Type Culture Collection and cultured according to their instructions [14].

### Macrophage infection and survival assay

Mice at 6–8 weeks of age were sacrificed by cervical dislocation. Femurs were obtained by using scissors to remove all muscle tissue from the bones. Bone marrow derived macrophages (BMDMs) were isolated from femurs by flushing with PBS using a 25-G needle as previously described [15]. Briefly, after 2 days, non-adherent BMDMs were collected and transferred at a density of 5 × 10^6^ cells/plate and were cultured for an additional 2–5 days. BMDMs were maintained in αMEM supplemented with 10 % endotoxin-free fetal bovine serum, 5 % CMG (conditioned medium containing M-CSF), penicillin, streptomycin, and glutamine. The RAW264.7 cells and BMDMs were plated in 24-well plates in complete tissue culture media without antibiotics at a concentration of 2.0 × 10^5^ cells per well and incubated overnight at 37 °C with 5 % CO_2_. The cells were infected with the *B. abortus* strain 2308 in triplicate wells at a multiplicity of infection (MOI) of 100:1 by centrifuging bacteria onto macrophages at 400 g for 10 min at 4 °C. Following 15 min of incubation at 37 °C in a 5 % CO_2_ atmosphere, the cells were washed three times with αMEM to remove extracellular bacteria and incubated for an additional 60 min in medium supplemented with 50 μg/ml gentamicin to kill extracellular bacteria. To monitor *B. abortus* intracellular survival, infected cells were lysed with 0.1 % Triton X-100 in phosphate-buffered saline (PBS), at certain time points, and serial dilutions of the lysates were rapidly plated onto tryptic soy agar plates to count the colony-forming units (CFUs) [16, 17].

### Production of lentivirus containing pre-miR-125b-5p or control Scr-miR

Based on the analysis using the Targetscan software (http://www.targetscan.org/), we found miR-125b-5p locus on chromosome 16: 77,644,273 to 77,648,343. The fragments of ∼ 400 bp encompassing the miR-125b-5p sequences acted as the sequences of pre-miR-125b-5p. Next, the genomic DNA from the RAW264.7 cells was used as a template (the forward primer: ACGCGTAGATCTCACTGCTCTTGCAGATCT, the reverse primer: ACGCGTGCGGCCGCTTGGAACAGTGACTTGCT), and the pre-miR-125b-5p was PCR-amplified and cloned into the MSCV PIG (Puro IRES GFP) retrovirus vector at the BglII and XhoI cloning sites. The pMSCV PIG-Scr-miR vector was constructed by annealing of Scr-miR oligonucleotide sequences containing the BglII and XhoI cloning sites, ligation of the annealed oligonucleotides into the MSCV PIG vector. The Scr-miR sequence was ACGTCTATACGCCCA. The generated plasmids were confirmed by DNA sequencing.

The lentivirus was produced by transiently transfecting HEK293T cells using the SuperFect transfection reagent (Qiagen, Redwood City, CA, USA) with three plasmid systems (pMSCV PIG-pre-125b-5p or pMSCV PIG-Scr-miR, psPAX2 and pMD2. G). The virus-containing supernatant was collected 72 h after transfection and filtered through a 0.45 mm filter (EMD Millipore, Billerica, MA, USA), and stored at −80 °C. Lentivirus titer on HEK293 cells was determined using the Adeno-X Rapid Titer kit (BD Biosciences Clontech, Palo Alto, CA, USA) according to the manufacturer’s instructions.

### Establishment of the RAW264.7 stable cell expressing miR-125b-5p or control Scr-miR

RAW264.7 cells were plated to 50 % confluency in a 12-well plate 24 h prior to viral infection. Next day, cells were infected with the miR-125b-5p or control Scr-miR lentivirus particles (the MOI was 10) overnight in antibiotics-free medium supplemented with 2 % FBS and Polybrene at a final concentration of 5 μg/ml. On the third day, the culture medium was replaced with 1 ml of complete medium (without Polybrene), and the cells were incubated for a further 48. After a 5-day infection, cells that had stably incorporated the lentiviral construct were selected by survival in the presence of puromycin (5 μg/mL). Afterwards, the medium was replaced with fresh puromycin-containing medium every 3–4 days, until resistant colonies were identified. Finally, several colonies were picked, expanded and assayed by RT-PCR analysis for stable miR expression.

### RNA quantification

RNA quantification was performed as previously described by Maegdefessel et al. [18]. Briefly, total RNA was isolated using a Trizol-based (Invitrogen, Carlsbad, CA, USA) RNA isolation protocol after RAW264.7 cells were infected with *B. abortus* at the indicated times. RNA was quantified by Nanodrop (Agilent Technologies, Santa Clara, CA, USA), and RNA and miRNA quality were evaluated using an Agilent 2100 Bioanalyzer (Agilent Technologies). First strand cDNA synthesis was performed for each RNA sample using the Superscript II RT Kit (Applied Biosystems, Waltham, MA, USA). Real time quantitative polymerase chain reaction was performed using TaKaRa PCR reagents (TaKaRa MiRNA qPCR Kit Ver. 2.0, TaKaRa Company, Kusatsu, Japan), the forward primer sequence for miR-125a-5p: AGTGTCCAATTTCCCAGAG, the forward primer sequence for miR-125b-5p: AGTGTTCAATCCCAGAG and the forward primer sequence for miR-351: GTCCGAGTTTCCCGAGGA. The reverse primers for miR-125a-5p, miR-125b-5p and miR-351 were universal adaptor primers designed and provided by the TaKaRa Company. The relative amount of miRNA was standardized against U6 snRNA, and the fold change for miRNA was calculated using the 2-DD computed tomography method compared with relative control.

### Transient modulation of miR-125b-5p Level in RAW264.7 cells

Knockdown of miR-125b-5p expression in the RAW264.7 cells was performed by transiently transfecting single-stranded anti–miR-125b-5p or control anti–miR oligonucleotides (Applied Biosystems), as previously described [6]. Briefly, 24 h before the experiment started, the culture media was changed and cells were maintained at 1 × 10^6^ cells/mL. Immediately before electroporation, the cells were washed twice in ice-cold PBS and 5 × 10^6^ cells/mL were re-suspended in 500 μL of serum-free medium with 100 nM of the relevant oligo (GGAGUUGGAUUGCUGAAUU) in 4-mm cuvettes. Subsequently, electroporation was performed using a Bio-Rad Gene Pulser (Bio-Rad Laboratories, Hercules, CA, USA) [19]. Mature miR-125b-5p expression in RAW264.7 cells was determined by real-time RT-PCR analysis (TaKaRa MiRNA qPCR Kit Ver. 2.0). The primer for the miR-125b-5p assay is shown in the method section 2.6.

### Western blot

Macrophages were lysed in ice-cold radioimmunoprecipitation assay (RIPA) buffer. The protein content was assayed by the BCA protein assay reagent (Pierce, Waltham, MA, USA). Twenty micrograms of protein were loaded into and size-separated by SDS–PAGE and then transferred to PVDF membrane. Afterwards, the membrane was incubated with a 1:1000 dilution of the primary antibody, followed by a 1:2000 dilution of horseradish peroxidase-conjugated secondary antibody. Protein bands were visualized by ECL (GE Healthcare, Chicago, Il, USA). The band densities were measured by densitometry (model GS-700, Imaging Densitometer; Bio-Rad), and the background was subtracted from the calculated area [20].

### Determination of NO production

The supernatant of each cell sample was collected at certain times point after the *B. abortus* challenge. Nitric oxide (NO) content was measured by analyzing its stable end product, nitrite, using the Griess reagent (Sigma-Aldrich Company, Saint Louis, MO, USA) as previously described [21]. Data are expressed as micromoles of nitrite (mean ± SEM).

### TNFα measurement

RAW264.7 cells were infected with *B. abortus* for the indicated times. The supernatants were collected for analyzing the secretion of TNFα using an enzyme-linked immunosorbent assay (ELISA) kit (eBioscience, San Diego, CA, USA) according to the manufacturer’s instructions [22].

### Immunofluorescent confocal microscopy

Immunofluorescent confocal microscopy visualization was performed as described previously [23]. Briefly, RAW264.7 macrophages were seeded in 8- or 16-well Permanox chamber slides (Costar Hatfield, PA, USA) and infected with GFP-expressing *B. abortus* for the indicated times. Following infection, cells were fixed with 1 % paraformaldehyde in PBS, permeabilized with 0.05 % saponin in PBS for 30 min at room temperature. After being blocked with antibody 2.4G2 for 1 h at room temperature, the slides were incubated with DAPI and the primary antibody (calnexin antibody) overnight at 4 °C. Subsequently, cells were washed with PBS and incubated for 1 h at room temperature with the fluorescein isothiocyanate (FITC)–conjugated secondary antibody (Alexa Fluor 568), as described in the Invitrogen protocol. Samples were visualized with a Zeiss LSM510 confocal microscope and the images were analyzed with the LSM confocal software [23].

### Statistical analysis

Statistical analysis was performed using the SPSS10.0 software (IBM Corporation, Armonk, NY, USA). Data are expressed as mean ± SEM. The statistical significance of the differences was evaluated by one-way ANOVA followed by the Student’s *t*-test. *P* < 0.05 was considered statistically significant [23].

## Results

### MiR-125b-5p is downregulated in *B. abortus*-infected macrophages

**Fig. 1:**
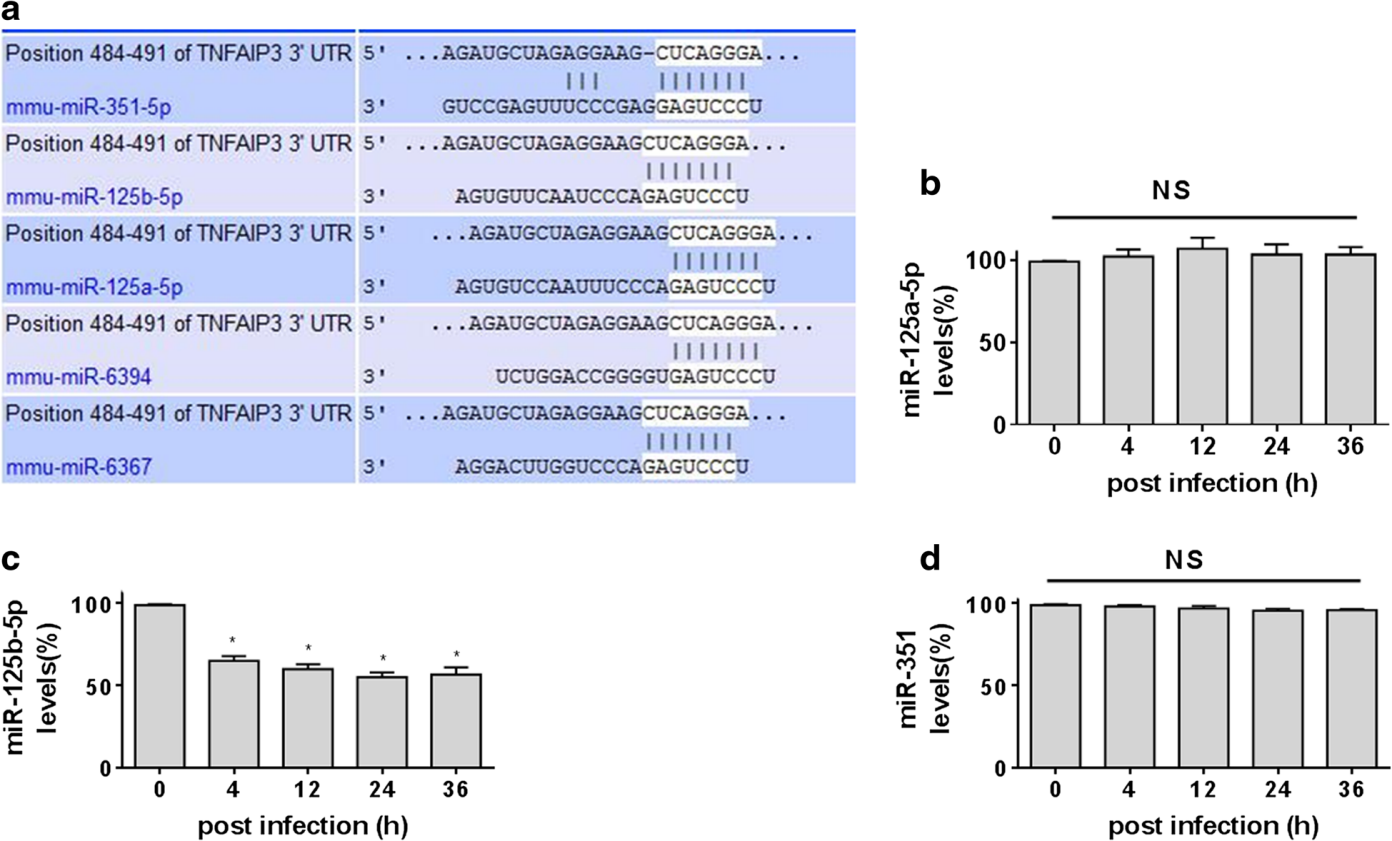
*B. abortus* infection induces downregulation of miR-125b-5p. **a** Prediction of miRNAs targeting 3′-UTR of A20 by Target Scan. **b**-**d** RAW264.7 macrophages were infected with *B. abortus* for the indicated times. RAW264.7 cell RNA was subjected to qRT-PCR analysis of miR-125a-5p **b**, miR-125b-5p **c** and miR-351 **d**. N is equal 3 in each group. Control group was defined as 100 %. RNA from resting RAW264.7 cells was used as control. *P < 0.05 VS control. NS indicates no significance

We previously reported that *B. abortus* infection enhanced the expression of A20 in macrophages, but the exact mechanism remains elusive. Considering that *Brucella* can establish chronic infection in part by regulating the expression of miRNAs [7], we hypothesized that the downregulation of a specific miRNA targeting A20 may be linked to the concomitant upregulation of A20 in *B. abortus*-infected macrophages. To investigate this hypothesis, we used TargetScan (http://www.targetscan.org/) to predict the intersection of target genes of miRNAs. Several miRNAs, including miR-351, miR-125a-5p, miR-125b-5p, miR-6394 and miR-6367 are predicted to potentially target A20, as shown in Fig. 1a. Given that miR-6394 and miR-6367 were not detected in the microRNA expression profile of *Brucella*-infected RAW264.7 cells [7], miR-351, miR-125a-5p and miR-125b-5p were chosen to test the biological relevance of the downregulation of these miRNAs during infection by *Brucella*. RAW264.7 cells were infected with *B. abortus* at a MOI of 100 and the miRNA expression was quantified. The RNA was isolated from infected macrophages over time and the fold change for miRNAs was calculated using the 2-DD computed tomography method after the relative amount of miRNA was standardized against the U6 snRNA. The expression of miR-125b-5p, but not miR-351 and miR-125a-5p, was decreased at 4 h with peak suppression at 24 h of *B. abortus* infection, as shown in Fig. 1b and d. This result indicates that the miR-125b-5p is specifically suppressed during *Brucella* infection. Actually, previous work has found that miR-125b-5p directly bind to the A20 gene 3′-UTR [6]. Therefore, the miR-125b-5p is likely to participate in the upregulation of the expression of A20 during *Brucella* infection.

### A20 is a target of miR-125b-5p

**Fig. 2:**
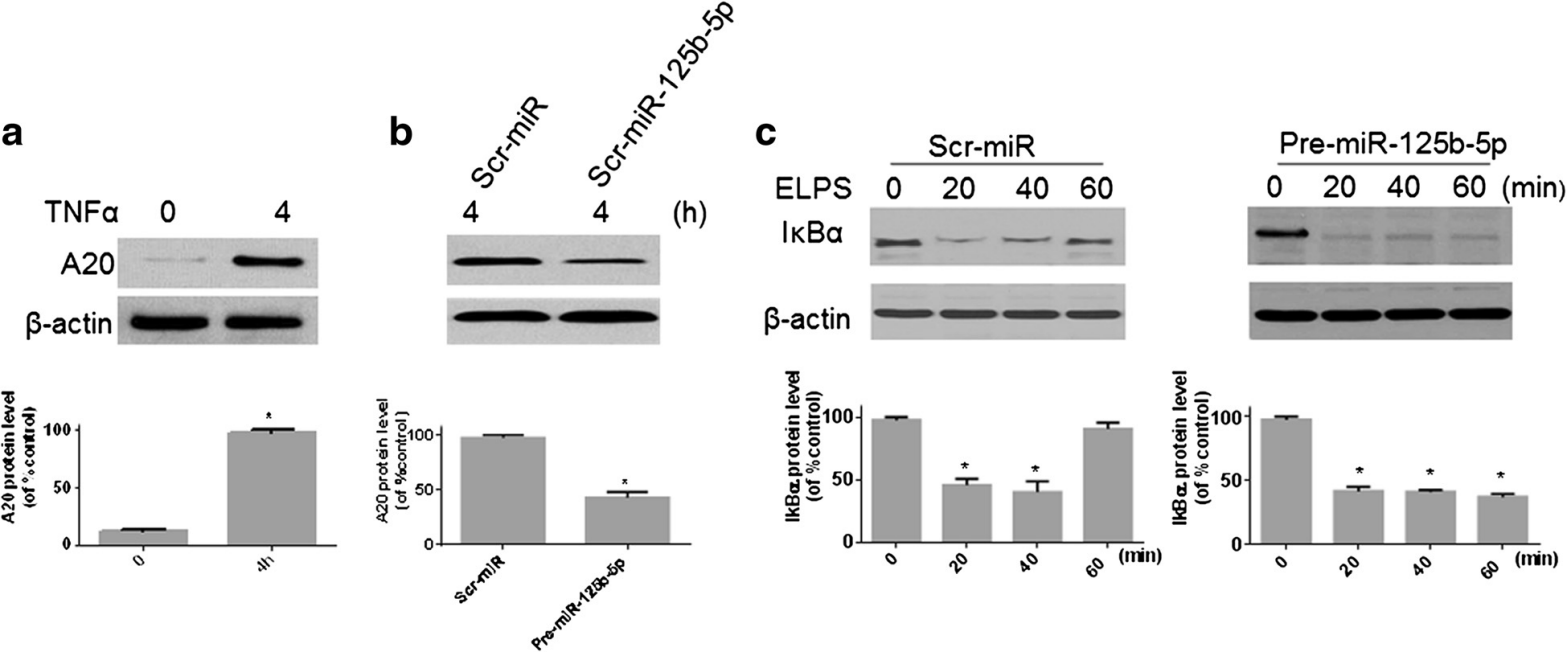
MiR-125b-5p downregulates its target A20. **a** RAW264.7 cells were treated with or without TNFα (10 ng/ml). **b** RAW264.7 cells, transduced with lentivirus containing scrambled microRNA (Scr-miR) or pre-miR-125b-5p, were treated with TNFα (10 ng/ml) for 4 h. Cell lysates **a** and **b** were subjected to immunoblotting analysis of A20. **c** RAW264.7 cells, transduced with lentivirus containing Scr-miR or pre-miR-125b-5p, were treated with 1000 ng/ml *E. coli* LPS (ELPS) for the indicated time points followed by immunoblotting of the cell lysates for IkBα and β-actin Western analysis. The blot is a representative of 3 independent experiments. The quantitative data for A20 and IkBα expression are shown under Western blotting data. Data are expressed as mean ± SEM (*n* = 3). The maximal ratio of IkBα or A20 expression /β-actin in the group was defined as 100 % (or control). *P < 0.05 VS control

As A20 is not expressed in the resting RAW264.7 cells, it is hard to test whether the miR-125b-5p is associated with upregulation of the A20 protein. To address this problem, the RAW264.7 cells were primed with 10 ng/ml of TNFα for 4 h to significantly increase the expression of A20 (Fig. 2a). In order to investigate whether A20 is a target of miR-125b-5p, RAW264.7 cells were infected with the lentivirus expressing pre-miR-125b-5p or with control scrambled microRNA (Scr-miR) and were treated with TNFα to induce the expression of A20. RAW264.7 cells infected with scr-miR upregulated A20 in response to TNFα, whereas pre-miR-125b-5p infected cells had a blunted response with significantly less A20 upregulation (Fig. 2b). To determine the functional consequences of the A20 regulation by miR-125b-5p, we evaluated NF-kB activation in response to the toll-like receptor ligands in cells expressing pre-miR-125b-5p [13]. RAW264.7 cells transfected with scr-miR or miR-125b-5p were treated with 1000 ng/ml *E. coli* LPS and NF-kB activation determined as measured by the degradation of IkBα [24]. IkBα was degraded at 20 and 40 min in response to LPS in both the Scr-miR and miR-125b-5p transfected cells; however, IkBα remained low at 60 min in the miR-125b-5p transfected cells, indicating persistent NF-kB activation (Fig. 1c). Taken together, these data suggest that A20 is a target of miR-125b-5p.

### *B. abortus* infection modulates the expression of A20 and NF-kB activity in a miR-125b-5p-dependent manner

**Fig. 3:**
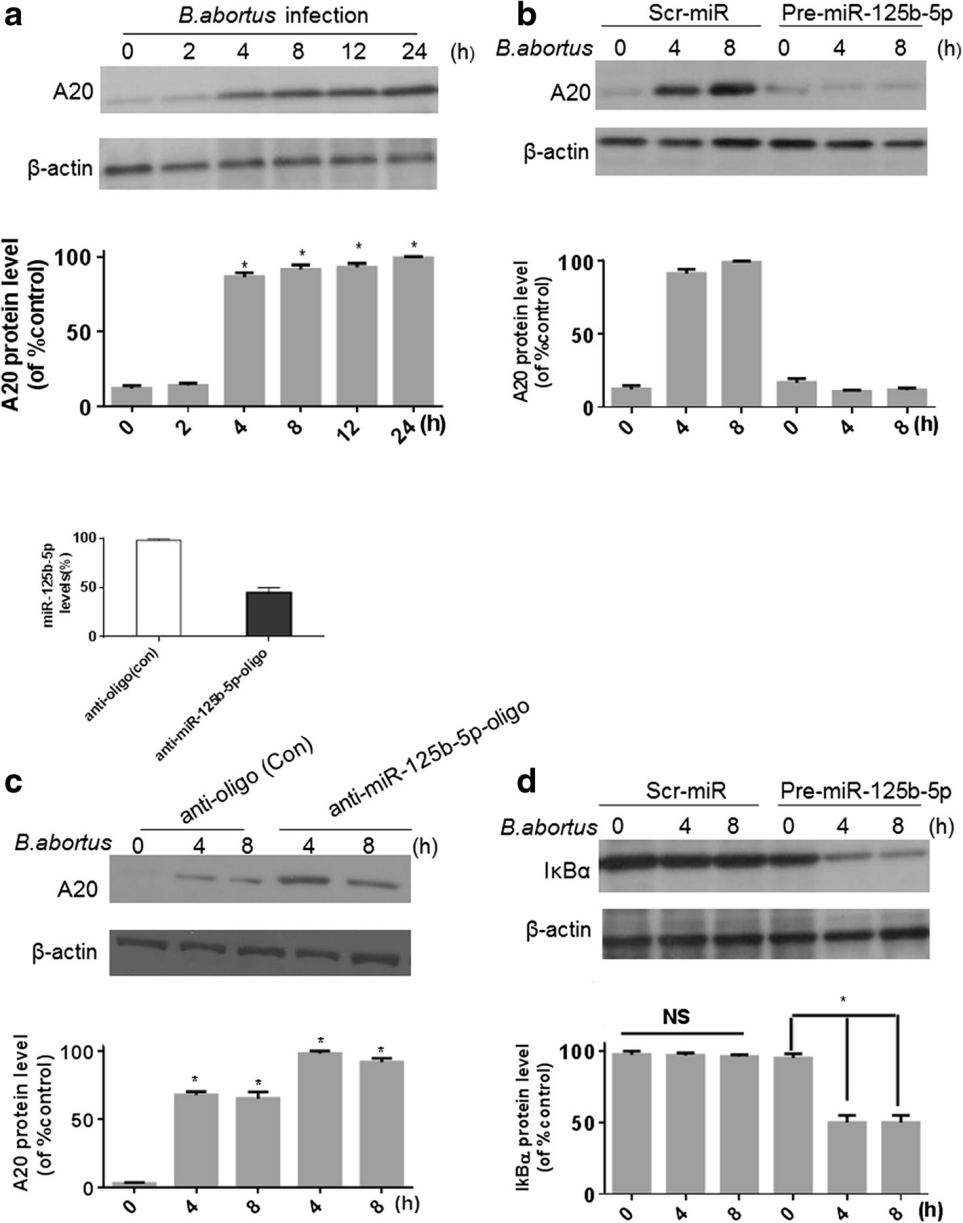
*B. abortus* infection modulates the expression of A20 and IkBα in a miR-125b-5p-dependent manner. **a** RAW264.7 cells were infected with *B. abortus* for different periods of time. **b** RAW264.7 cells were transfected with either Scr-miR or Pre-miR-125b-5p and infected with *B. abortus* for 4 or 8 h. Expression of A20 in **a** and **b** were determined by immunoblotting with A20 antibody. **c** RAW264.7 cells transfected with control anti-oligonucleotides (anti-oligo (Con)) or anti-miR-125b-5p oligonucleotides (anti- miR-125b-5p-oligo) were infected with *B. abortus* and their RNA was subjected to qRT-PCR analysis of miR-125b-5p (top panel). A20 expression in cell lysates was measured by immunoblotting with A20 antibody. **d** RAW264.7 cells transfected with Scr-miR or Pre-miR-125b-5p were infected with *B. abortus* for the indicated time periods, and IkBα expression was monitored in RAW264.7 cell lysates with IkBα antibody. The blot is representative of 3 independent experiments. Quantitative data for A20 and IkBα expression are shown under the Western blotting data. Data are expressed as mean ± SEM (*n* = 3). The maximal ratio of IkBα or A20 expression /β-actin in the group was defined as 100 % (or control). *P < 0.05 VS control. NS indicates no significance

To test whether *B. abortus* infection induces downregulation of miR-125b-5p to regulate the expression of A20, we evaluated the expression of A20 in *B. abortus* infected-RAW264.7 cells. The results revealed that A20 was markedly upregulated after 4 h post infection and remained elevated for 24 h, as shown in Fig. 3a. RAW264.7 cells were transduced with the lentivirus containing pre-miR-125b-5p or Scr-miR prior to *B. abortus* infection. It was found that A20 expression was decreased significantly in infected cells expressing pre-miR-125b-5p compared to cells expressing control Scr-miR (Fig. 3b). These results indicate that miR-125b-5p regulates A20 expression in *B. abortus*-infected macrophages. In order to further confirm the direct involvement of miR-125b-5p on the regulation of A20 expression, we also transfected cells with anti-miR-125b-5p oligonucleotides to knock down the miR-125b-5p levels prior to infection with *B. abortus* (Fig. 3c, top panel). Following infection of the RAW264.7 cells, A20 levels were increased significantly compared to cells transfected with control anti-oligo (Fig. 3c, second panel), supporting the argument that miR-125b-5p is directly linked to the regulation of A20 expression in *B. abortus*-infected cells.

Since A20 is a feedback inhibitor of NF-kB signaling [24], the *B. abortus* infection-induced downregulation of miR-125b-5p should inhibit NF-kB activation. To test this notion, cells transfected with a pre-miR-125b-5p or Scr-miR were infected with *B. abortus* and analyzed. Specifically, NF-kB activation was measured by monitoring the degradation of IkBα. The results revealed that compared to cells transfected with control Scr-miR, IkBα was significantly degraded in RAW264.7 cells transfected with pre-miR-125b-5p indicating, as shown in Fig. 3d, that the miR-125b increased NF-kB activity. Taken together, these data demonstrate that *B. abortus* infection modulates the expression of A20 and NF-kB activity in a miR-125b-5p-dependent manner.

### *B. abortus*-induced downregulation of miR-125b-5p is linked to *B. abortus* intracellular growth via regulating macrophage activation

**Fig. 4:**
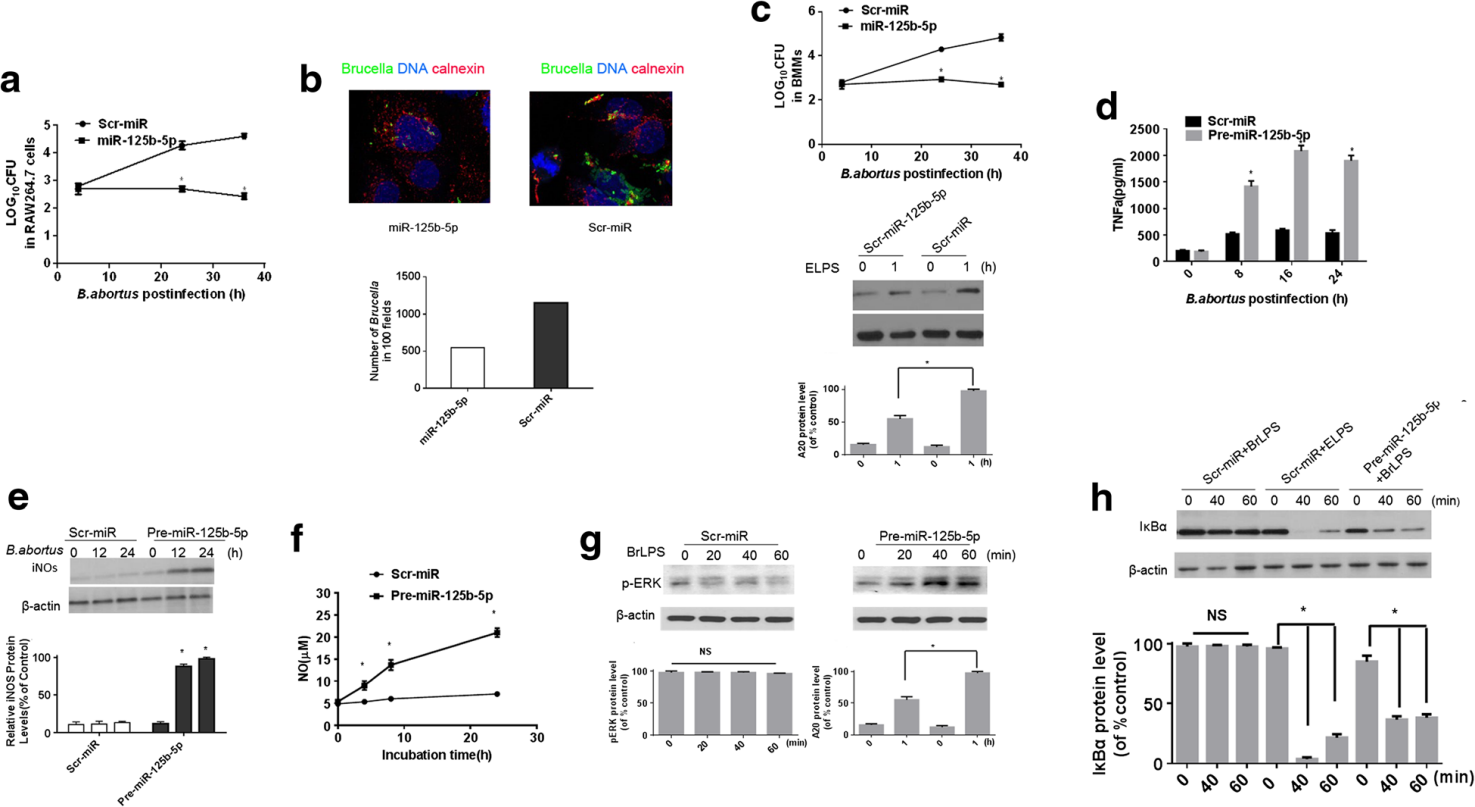
MiR-125b-5p mediates intracellular *B. abortus* replication via the suppression of macrophages activation. **a** RAW264.7 cells transduced with Scr-miR or Pre-miR-125b-5p were infected with *B. abortus* for the indicated time periods. *B. abortus* intracellular growth was determined by CFUs assay. The group containing Scr-miR was defined as control. *P < 0.05 vs control. Data are expressed as mean ± SEM. **b** RAW264.7 cells, transduced with Scr-miR or Pre-miR-125b-5p, were infected with *B. abortus* for 36 h, immunofluorescence analysis of the infected cells was performed. The number of *Brucella* in 100 fields is shown under the confocal microscopy images. Green: *Brucella*; blue: cell nucleus; red: calnexin (scale bar = 20 um). **c** BMDMs transduced with Scr-miR or Pre-miR-125b-5p was infected with *B. abortus* for the indicated time periods. *B. abortus* intracellular growth was determined by the CFUs assay. The group containing Scr-miR was defined as control. *P < 0.05 vs control. Data are expressed as mean ± SEM. In the middle panel of **c**, BMDMs transduced with lentivirus containing scrambled microRNA (Scr-miR) or pre-miR-125b-5p, were treated with ELPS (1000 ng/ml) for 1 h. Cell lysates were subjected to immunoblotting analysis of A20. **d**, **e** and **f** RAW264.7 cells, transfected with Scr-miR or Pre-miR-125b-5p, were infected with *B. abortus* for 8, 16 and 24 h, respectively, TNFα (**d**) or NO production in the supernatants of cells was measured by ELISA (**f**), and iNOS protein expression in the cells was determined by immunoblotting with iNOS antibody (**e**) **g** and **h** RAW264.7 cells, transfected with Scr-miR or Pre-miR-125b-5p, were stimulated with 10 μg/ml *Brucella* LPS (BrLPS) or 1000 ng/ml ELPS for the indicated time (min). pERK (**g) ** or IkBα ( **h**) expression was determined using antibodies specific for pERK or IkBα. Data are representative of three independent experiments. Quantitative data for IkBα, pERK and iNOS expression are shown under the Western blotting data. The maximal ratio of IkBα, pERK or iNOS expression /β-actin in the group was defined as 100 % (or control). *P < 0.05 VS control. NS indicates no significance

Having shown that *B. abortus* infection induces the suppression of miR-125b-5p and induction of A20 expression, we further investigated the role of miR-125b-5p in *B. abortus* infection. To ascertain the role of miR-125b-5p in bacterial intracellular survival, the *B. abortus* CFUs were counted in infected RAW264.7 cells after transfection with either Scr-miR or Pre-miR-125b-5p. The results indicate, as evident in Fig. 4a, that survival of *B. abortus* was significantly decreased in the presence of miR-125b-5p compared to Scr-miR. To assess the bacterial burden, confocal microscopy was also used to examine the intracellular bacteria colocalization with the endoplasmic reticulum (ER) marker calnexin at 36 h post infection. Consistent with the CFU assay, the number of *B. abortus* in the ER was markedly decreased in the RAW264.7 cells transfected with pre-miR-125b-5p (Fig. 4b). Additionally, BMDMs transfected with pre-miR-125b-5p were used to confirm the role of miR-125b-5p in mediating *Brucella* intracellular growth. Our data showed that the regulatory role of miR-125b-5p in BMDMs was similar to that observed in RAW264.7 cells (Fig. 4c). Collectively, our results suggest that the downregulation of miR-125b-5p facilitates the survival of *B. abortus* in macrophages.

We next examined the mechanism by which the downregulation of miR-125b-5p induced by *B. abortus* facilitates its intracellular replication. Since activated macrophages are the primary source for *Brucella* elimination in the infected host [3, 25, 26], we hypothesized that miR-125b-5p mediates bacterial intracellular growth by regulating macrophages activation. Considering that the increase of TNFα production is the hallmark of activated macrophages [27], TNFα production was examined after RAW264.7 cells were infected with *B. abortus*. The results showed that TNFα was markedly increased in the presence of pre-miR-125b-5p compared to Scr-miR in response to *B. abortus* infection, as shown in Fig. 4c. To further confirm that miR-125b-5p regulates macrophage activation after infection with *Brucella*, we also evaluated iNOS expression, NO production, and activation of ERK and NF-kB as additional markers of macrophages activation [3, 28, 29]. iNOS expression (Fig. 4d) and NO production (Fig. 4e) were prominently increased in the presence of pre-miR-125b-5p compared to Scr-miR after *B. abortus* infection indicating that there is increased macrophage activation.

*Brucella* LPS (BrLPS) is generally reported to be a poor inducer of inflammatory cytokines and macrophage activation [1, 30]. To determine whether miR-125b-5p regulates ERK activation, RAW264.7 cells expressing Scr-miR or pre-miR-125b-5p were stimulated with BrLPS and ERK activation was examined. The results revealed that ERK was significantly enhanced in pre-miR-125b-5p cells compared to Scr-miR cells (Fig. 4f and g). Additionally, we also observed that stimulation of cells with BrLPS in the presence of pre-miR-125b-5p triggered an increase of NF-kB activity similar to that observed with the more potent *E. coli* LPS stimulation (Fig. 4g). Taken together, these data indicate that *B. abortus*-induced downregulation of miR-125b-5p mediates *B. abortus* intracellular growth via suppression of macrophages activation.

## Discussion

*Brucella* has the ability to subvert the innate immune defense of host macrophages in order to establish infection within the macrophages. *Brucella* may establish *Brucella*-containing vacuole (BCV) for its intracellular growth by regulating the expression of miRNAs [7]. However, no studies have focused on how the utilization of the miRNAs during *Brucella* infection influences bacterial survival. Our data have revealed that the miR-125b-5p is downregulated in *B. abortus* infection. Focusing on miR-125b-5p, we have demonstrated that its downregulation is associated with the concomitant upregulation of one of its target, namely A20, which mediates *B. abortus* intracellular replication via the regulation of NF-kB activity and macrophage activation.

Considering that NF-kB is a prominent regulator of macrophage activation [31], we focused our investigation on the putative miR-125b-5p target A20, which is an important regulator of NF-kB activation. The upregulation of A20 in *B. abortus*-infected macrophages was decreased in the presence of a miR-125b-5p and increased when cells were transfected with anti-miR-125b-5p, suggesting that the *B. abortus*-induced suppression of miR-125b-5p is linked to the enhanced expression of A20. At the same time, transfection of the pre-miR-125b-5p was associated with increased NF-kB activity, suggesting that regulation of A20 by miR-125b-5p likely mediates NF-kB activity during *B. abortus* infection.

We have reported that A20 promotes *B. abortus* intracellular growth via inhibition of macrophage activation and their death. Our observations now add a further layer of complexity to this fine-tuning of macrophage activation by adding a miRNA-dependent mechanism of regulation of A20. Accordingly, we propose that the *B. abortus*-induced suppression of miR-125b-5p leads to increased A20 expression and concomitant reduction of macrophage activation via suppression of TNFα, which is required for macrophage activation [27]. The observed suppression of nitrite production likely facilitates the survival of *Brucella* in macrophages [28]. This hypothesis is also supported by our data that transfection of the miR-125b-5p prior to infection is associated with ERK activation, another marker of macrophage activation, which is consistent with the findings in TREM-2 knock-down macrophages [2]. Our results demonstrate an unappreciated role of miR-125b-5p downregulation in favoring *B. abortus* intracellular survival.

In conclusion, this study provides reference points for further investigation into understanding the mechanism of *Brucella* intracellular survival mediated by miRNAs. The data in *Brucella*-infected macrophages support the notion that miRNA may play an important role in favor of *Brucella* survival. It is tempting to speculate that targeted intervention of essential miRNA could provide an attractive therapy for the management of Brucellosis.

## Conclusions

The following conclusions can be drawn from our results: 1) MiR-125b-5p is downregulated in *Brucella*-infected macrophages; 2) *Brucella* infection suppresses NF-kB activity in a miR-125b-5p-dependent manner; 3) MiR-125b-5p inhibits *Brucella* –infected macrophage activation.

## Abbreviations

BCV, *Brucella*-containing vacuole; BMDMs, Bone marrow derived macrophages; BrLPS, *Brucella* LPS; CFUs, the colony-forming units; CMG, conditioned medium containing M-CSF; ELISA, Enzyme-linked immunosorbent assay; ER, endoplasmic reticulum; FITC, fluorescein isothiocyanate; miRNAs, microRNAs; MOI, multiplicity of infection; RIPA, radioimmunoprecipitation assay; Scr-miR, scrambled microRNA
